# Glutaric aciduria type 1 presenting with Parkinsonism with presynaptic dopaminergic dysfunction in an adult female: a case report

**DOI:** 10.1007/s10048-026-00940-0

**Published:** 2026-09-15

**Authors:** Matej Lokar, Katja Jarc Georgiev, Tomaž Rus, Nadan Gregorič, Borut Peterlin, Maruša Škrjanec Pušenjak, Dejan Georgiev

**Affiliations:** 1 Department of Neurology, University Medical Centre Ljubljana, Ljubljana, Slovenia; 2 Faculty of Medicine, University of Ljubljana, Ljubljana, Slovenia; 3 Clinical Institute for Genomic Medicine, University Medical Centre Ljubljana, Ljubljana, Slovenia; 4 Center for Rare Diseases, University Children’s Hospital, University Medical Centre Ljubljana, Ljubljana, Slovenia; 5 Department of Nuclear Medicine, University Medical Centre Ljubljana, Ljubljana, Slovenia; 6 Department of Endocrinology, Diabetes and Metabolic Diseases, Division of Intern al Medicine, University Medical Centre Ljubljana, Ljubljana, Slovenia; 7 Artificial Intelligence Laboratory, Faculty of Computer and Information Science, University of Ljubljana, Ljubljana, Slovenia

**Keywords:** Glutaric Aciduria Type I, *GCDH* Protein, Parkinsonian Disorders, Dopamine Transporter Imaging, Levodopa, Inborn Errors of Metabolism

## Abstract

Glutaric aciduria type 1 (GA1) is an autosomal recessive neurometabolic disorder caused by pathogenic variants in the *GCDH* gene, typically presenting in infancy with dystonia following encephalopathic crisis. Late- and adult-onset forms are rare and may manifest with nonspecific neurological features. We describe a 43-year-old woman with levodopa-responsive parkinsonism and presynaptic dopaminergic dysfunction. Genetic analysis revealed compound heterozygous *GCDH* variants, consistent with GA1. One of the reported variants (c.1178G > A, p. Gly393Glu) is extremely rare and has never been associated with adult-diagnosed GA1 with atypical features. Furthermore, this is the first report of GA1 patient presenting with parkinsonism in whom we demonstrated presynaptic dopaminergic dysfunction, along with a favorable response to levodopa.

**Supplementary Information:**

The online version contains supplementary material available at https://doi.org/10.1007/s10048-026-00940-0.

## Introduction

Glutaric aciduria type 1 (GA1, OMIM#231670) is an autosomal recessive neurometabolic disorder caused by deficiency of glutaryl-CoA dehydrogenase (GCDH) involved in the degradation pathway of lysine, hydroxylysine and tryptophan. Pathogenic *GCDH* variants (chromosome 19p13.13) lead to the accumulation of glutaric acid, 3-hydroxyglutaric acid, glutaconic acid and glutarylcarnitine, resulting in mitochondrial dysfunction, excitotoxicity and disrupted neurotransmission [1].

Newborns with GA1 are typically asymptomatic, although macrocephaly may be present and predisposes to stretching of bridging veins, subdural hematomas and arachnoid cysts [1, 2]. Catabolic states may precipitate an acute encephalopathic crisis with bilateral striatal injury and consequent irreversible complex movement disorder with predominant dystonia (acute-onset GA1). With age, dystonia becomes more fixed and may be associated with parkinsonism and spasticity [1]. In contrast, the insidious-onset GA1 is characterized by gradual neurological deterioration without a clear encephalopathic crisis, while the late-onset forms present after six years of age with heterogeneous and often nonspecific features including cognitive dysfunction, headaches, vertigo, gait ataxia, pyramidal signs, seizures, incontinence, peripheral neuropathy and psychiatric disorders [1, 3]. Neuroimaging findings in late-onset GA1 differ from classical striatal lesion seen in the acute-onset GA1 and might show intact striatum with subependymal nodules, periventricular abnormalities and frontotemporal hypoplasia [4]. Based on the urinary glutaric acid concentrations, the patients can be subdivided into low and high excretors [1].

We report an adult female patient with rare compound heterozygous *GCDH* variants, presenting with levodopa-responsive parkinsonism associated with presynaptic dopaminergic dysfunction, thereby expanding the genotype-phenotype spectrum of GA1.

## Case presentation

43-year-old woman, a mother of two healthy children, was referred to the Department of Neurology for evaluation of recurrent headaches accompanied by transient blurry vision and diplopia. Pregnancy and birth history were unremarkable. During infancy, she experienced pneumonia with no documented metabolic crisis. Later in infancy, she sustained a head hemorrhage following a fall from a stroller. The speech and motor developmental delays that developed afterwards were attributed to this head injury. Despite these challenges, the patient completed higher education, maintained full-time employment and remained functionally independent. Family history was negative for neurological or metabolic disorders.

On examination, she was mildly dysarthric. The oculomotor examination was unremarkable. Mild dystonic posturing of the upper extremities and mild bradykinesia, more pronounced in the legs and on the left side, were observed, along with bilateral fine motor impairment. The muscle tone was mildly rigidly increased in the lower extremities, and the gait was slightly slower. The plantar responses were flexor. There were no hypomimia, tremor or postural instability present. (Online resource 1 – Supplementary video). No clinically relevant non-motor symptoms were reported by the patient.

**Fig. 1:**
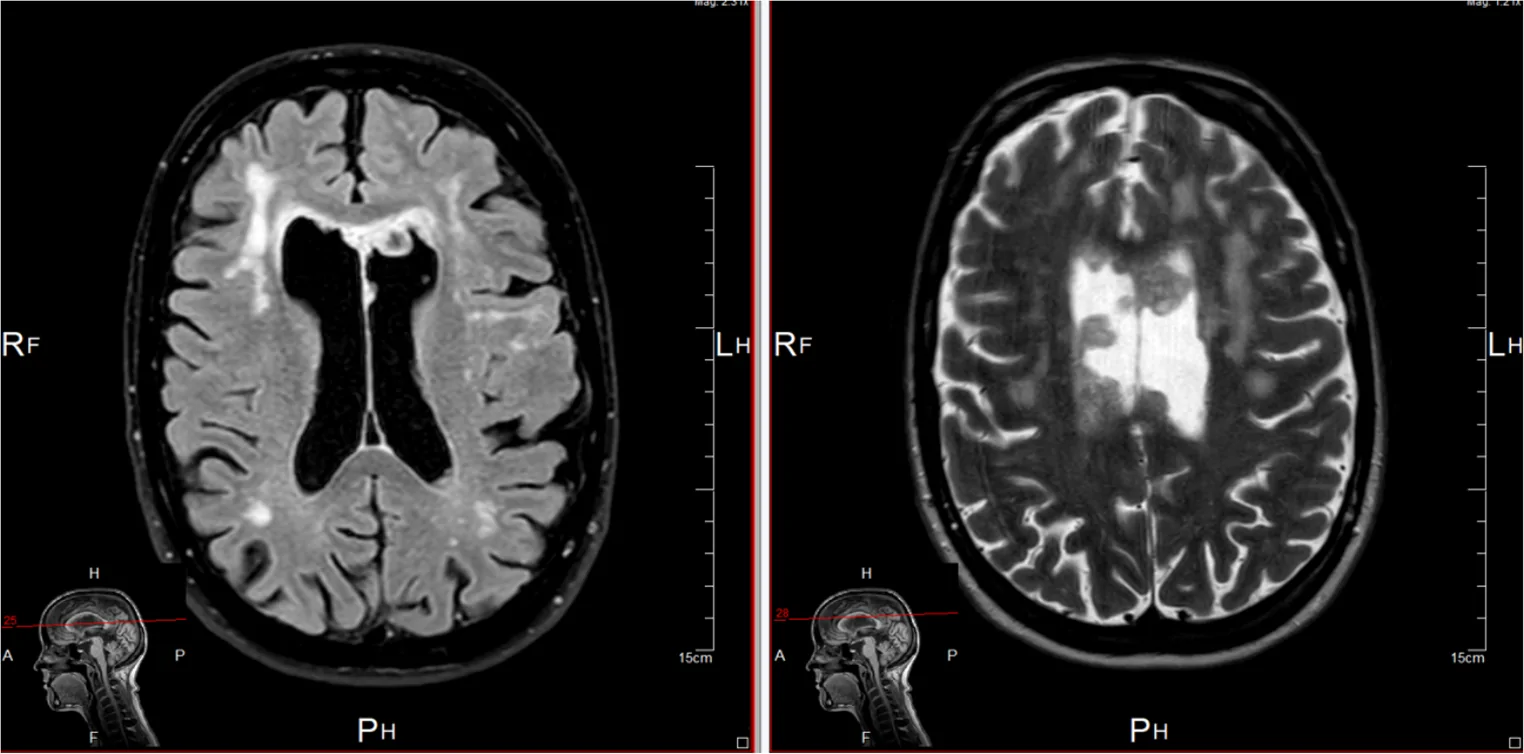
Brain MRI shows multiple non-enhancing subependymal nodules in both lateral ventricles and periventricular leukoencephalopathy, there are no signs of hydrocephalus

**Fig. 2:**
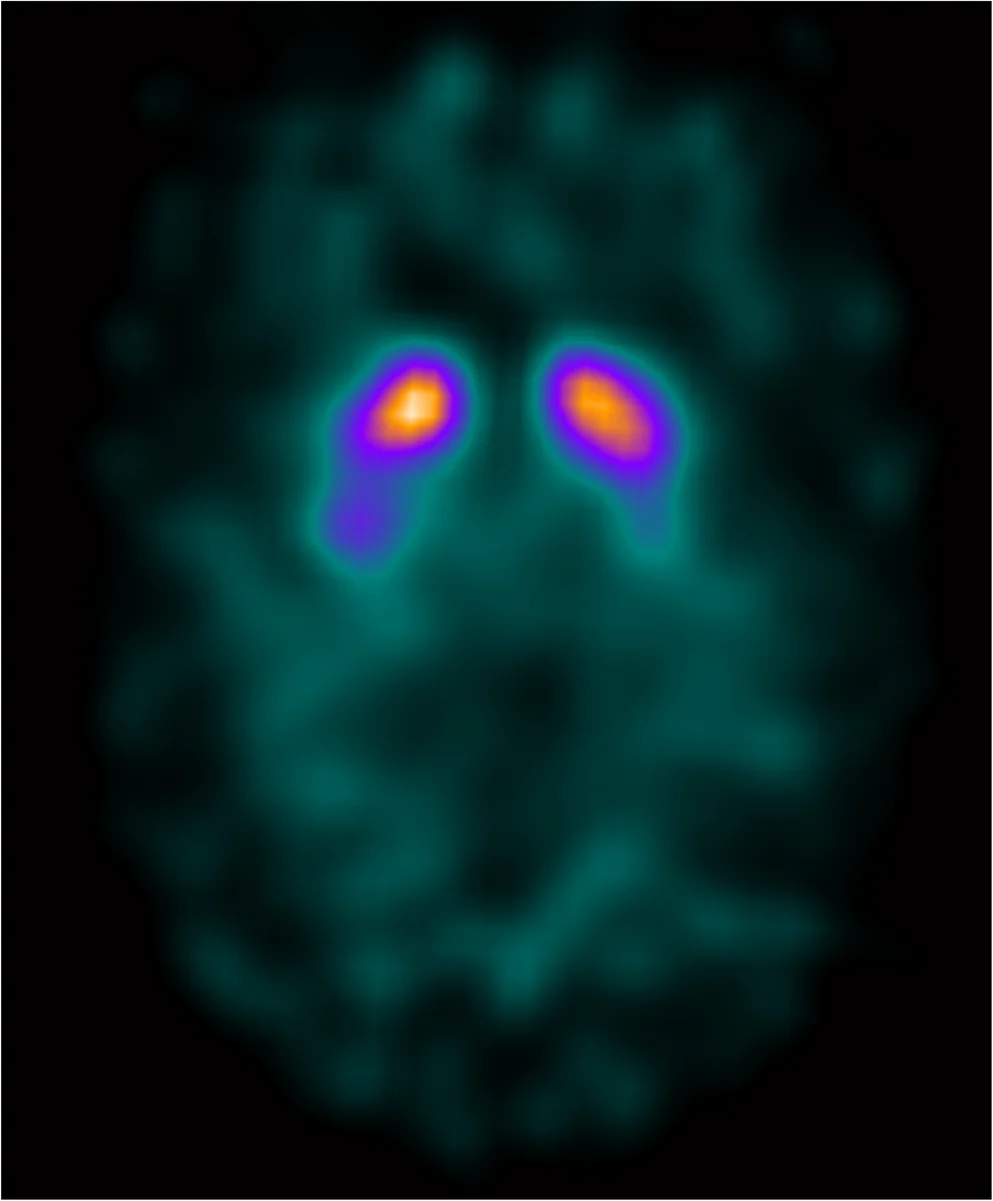
Dopamine transporter SPECT showing presynaptic dopaminergic impairment. Quantitative analysis showed preserved DAT binding in the caudate nuclei (z = 0.47 on the right and z = − 0.50 on the left) but marked bilateral reductions in the anterior putamina (z = − 2.5 bilaterally) and more profound reductions in the posterior putamina (z = − 3.5 on the right and z = − 4.2 on the left). The deficit was relatively symmetric, with asymmetry z-scores of 1.59 for the striatum and − 0.26 for the putamen

Electroencephalography and ophthalmological examination were unremarkable. Brain MRI with contrast revealed multiple non-enhancing subependymal nodules in both lateral ventricles and periventricular leukoencephalopathy, with no apparent striatal lesion and no hydrocephalus (Fig. 1). The whole-spine MRI was unremarkable. Neuropsychological assessment revealed psychomotor slowing and isolated executive dysfunction, with accompanying deficits in visuospatial abilities and memory. Further genetic and biochemical analysis was consistent with the diagnosis of GA1 (see below). Dopamine transporter SPECT (DAT-SPECT) demonstrated bilateral presynaptic dopaminergic dysfunction, predominantly affecting the posterior putamina (Fig. 2). Despite the mild signs of parkinsonism, a levodopa challenge test resulted in a 50% improvement in MDS-UPDRS motor score (OFF = 12, ON = 6). Treatment with low dose levodopa was initiated. At 18 months levodopa/benserazide 100/25 mg twice daily showed sustained benefit without fluctuations, dyskinesias, or other adverse effects. She was also referred to an adult metabolic specialist, who recommended a low-protein and low-lysine diet with carnitine supplementation and to increase the caloric intake during catabolic stress.

### Genetic and biochemical analysis

Whole exome sequencing of the defined clinical target was performed on the isolated DNA sample. The fragmentation and enrichment of the isolated DNA sample were performed according to the protocol TwistExomev5_0_CNV (Twist Bioscience, South San Francisco, CA, USA). Next-generation sequencing was performed with Illumina NovaSeq 6000 (Illumina, San Diego, CA, USA). After duplicates were removed, the alignment of reads to UCSC hg19 reference assembly was done using BWA algorithm (v0.6.3) and variant calling was done using GATK framework (v2.8). Only variants exceeding the quality score of 30.0 and depth of 5 were used for down-stream analyses. Variant annotation was performed using ANNOVAR and snpEff algorithms, with pathogenicity predictions in dbNSFPv2 database. Reference gene models and transcript sequences are based on RefSeq database. Structural variants were assessed using CONIFER v0.2.2 algorithm. Variants with population frequency exceeding 1% in 1000 genomes and ESP6500, synonymous variants, intronic variants and variants outside the clinical target were filtered out during analyses. The interpretation of sequence variants was based on ACMG/AMP standards and guidelines [5]. Because of the findings of subependymal nodules, the analysis focused on genes associated with tuberous sclerosis, in addition to hereditary meningeomas, hydrocephalus and extended panel of genes for monogenic diseases with signs of cerebral palsy. Mean coverage of target regions was 154x. WES revealed compound heterozygous variants in the *GCDH* gene (trans configuration based on manual inspection of the reads on IGV); a known pathogenic (NM_000159.4, c.1204 C > T, p.Arg402Trp) [PM2, PS3, PM5, PP3] and a rare, likely pathogenic variant (NM_000159.4, c.1178G > A, p.Gly393Glu) [PM2, PM3, PP3, PP4] [5]. Subsequent dried blood spot analysis by liquid chromatography-tandem mass spectrometry demonstrated elevated glutarylcarnitine (C5DC, 2.5-fold above the upper limit of normal [ULN]), together with increased of C5DC/octanoylcarnitine, C5DC/palmitoylcarnitine and C5DC/free carnitine (25-28-fold above the ULN). Free carnitine concentration was markedly reduced (3.38 µmol/L; reference range 8.44–57.81 µmol/L). These findings were consistent with GA1. Urinary organic acid analysis by gas chromatography–mass spectrometry (GC-MS), performed using a semiquantitative method, demonstrated markedly increased glutaric acid, 3-hydroxyglutaric acid, and glutaconic acid, consistent with a high-excretor phenotype. Plasma amino acid analysis by LC-MS/MS was also performed, and all measured amino acid concentrations were within their respective reference ranges.

## Discussion

This report describes a rare case of GA1 presenting with parkinsonism diagnosed in adulthood. Despite the history of developmental delay and intracranial hemorrhage in infancy, the absence of metabolic crisis and nonspecific findings delayed the time to diagnosis [2]. Only after unspecific complaint of headache and double vision, neuroimaging findings prompted genetic testing, highlighting the diagnostic challenges associated with GA1.

GA1 diagnosed in adulthood is occasionally referred to as adult-onset GA1, with only nine such cases reported in the literature so far [3]. Whether these patients represent a distinct clinical phenotype or a continuum of late-onset disease characterized by milder symptoms and slower progression remains unresolved [1, 3]. In the present case, non-specific neurological symptoms were already evident in early childhood, suggesting partial overlap with insidious-onset GA1. However, the absence of prominent dystonia, lack of striatal injury on neuroimaging, and presence of atypical clinical features are more consistent with late-onset disease phenotype and likely contributed to a substantial delay in establishing the correct diagnosis.

Similar cases of GA1 misdiagnosed in childhood and later presenting in adulthood with complex movement disorder presentation, including chorea, dystonia and myoclonus have been reported [6]. Our patient exhibited a milder phenotype with mild parkinsonian features and subtle dystonic posturing, further complicating the diagnostic process. Additionally, although several cases have been reported where abnormal acylcarnitine profiles at newborn screening (NBS) in an unaffected neonate revealed previously unrecognized maternal GA1 [7, 8], our patient’s children had normal profiles at NBS. This case therefore highlights the diagnostic challenges associated with mild GA1 phenotypes.

Our principal differential diagnosis was coincidental Parkinsons disease. As parkinsonism is a recognized feature of GA1 [9], because of the relatively young age (43 years), lack of any non-motor features and the characteristic MRI abnormalities associated with GA1, this diagnosis is unlikely. Furthermore, the patient reported no family history of parkinsonism or related neurological disorders that might suggest an alternative genetic cause. Finally, the genetic panel included numerous genes associated with monogenic Parkinson’s disease (e.g., *DNAJC6*,* PARK7*,* PINK1*,* PRKN*,* SNCA*, and *VPS13C*), and no relevant pathogenic variants were identified.

Movement disorders in adults with GA1 are complex and not thoroughly characterized. In a review of 53 late-onset cases, only 7 of 20 symptomatic adults had movement disorders features, as shown in Table 1 [10]. Although parkinsonism in adults with GA1 is typically described as akinetic-rigid syndrome [9], asymmetric tremor was the only parkinsonian sign reported, while bradykinesia, rigidity, and postural instability were not described [11–14]. Parkinsonism in GA1 patients therefore requires further characterization.

**Table 1:** Summary of published movement disorders presentation in glutaric aciduria type 1 patients diagnosed in adulthood

Author	Age at diagnosis	Movement disorders presentation
Sitta et al. 2021 [11]	18 years	Tremor
Sitta et al. 2021 [11]	26 years	Dystonia
Ulamanová et al. 2020 [6]	45 years	Dysarthria, generalized chorea, possible myoclonus and dystonia
Ulamanová et al. 2020 [6]	45 years	Cervical dystonia, orofacial dystonia, dysarthria, chorea, possible myoclonus, task specific hand dystonia (during writing and tapping), dystonic posturing of the feet
Prevett et al., 1996 [15]	50 years	Dysarthria, lingual dystonia, orofacial dyskinesia, dystonic posturing of both hands
Harting et al. 2009 [12]	65 years	Hand tremor
Külkens et al. 2005, Boy et al. 2017 [13, 14]	66 years	Orofacial dyskinesia, asymmetric hand tremor, ataxia
Boy et al. 2017 [14]	77 years	Tremor, dysdiadochokinesis

To our knowledge, this is the first report demonstrating presynaptic dopaminergic dysfunction in GA1. We found no reports of DAT-SPECT being undertaken in other GA1 patients. Our patient experienced 50% improvement on MDS-UPDRS, despite the initial motor score being low. Responsiveness to levodopa suggests preserved postsynaptic dopaminergic pathways and indicates potential benefit from dopaminergic therapy in selected patients [16–18]. Presynaptic dopaminergic dysfunction in GA1 may result from chronic neuronal damage. Nigrostriatal dopaminergic terminals in the striatum may be affected by long-term cerebral accumulation of glutaric and 3-hydroxyglutaric acid [19], as these metabolites have been suggested to impair mitochondrial function, promote oxidative and excitotoxic injury, and disturb metabolic interactions between astrocytes and neurons [20–22]. Additionally, white matter abnormalities, potentially reflecting impaired myelination and blood-brain barrier dysfunction, might also lead to nigrostriatal axon injury [12, 23–25].

Likely pathogenic *GCDH* variant p.Gly393Glu has previously been reported only once in a male patient with early disease onset following an acute metabolic crisis, presenting with predominantly dystonic phenotype and bilateral caudate lesion on imaging [26]. Our patient, however exhibited a milder, insidious course presenting with parkinsonism, and accompanied by atypical imaging findings, thereby broadening the phenotypic features associated with this likely pathogenic *GCDH* variant.

In summary, we report a novel observation of levodopa-responsive parkinsonism and presynaptic dopaminergic dysfunction in a GA1 patient diagnosed in adulthood, carrying a rare, likely pathogenic GCDH variant, and exhibiting a subtle, longstanding neurological manifestation. This report expands the phenotypic spectrum of an extremely rare GA1 genotype.

## Supplementary Information

Below is the link to the electronic supplementary material.

Supplementary Video 1 Video demonstrating the neurological examination. The initial segment shows the general neurological assessment, where mild dysarthria can be observed. The second segment shows the ON-medication MDS-UPDRS assessment demonstrating mild bradykinesia, especially pronounced in the left lower extremity, mild dystonic posturing of the left upper extremity, and slightly slowed gait. There are no facial hypomimia, tremor or postural instability present. (MP4 179 MB)

## Data Availability

No datasets were generated or analysed during the current study.
